# 15-item Roland-Morris Disability Questionnaire (RMDQ-15): structural and criterion validity on patients with chronic low back pain

**DOI:** 10.1186/s12891-022-05953-y

**Published:** 2022-11-12

**Authors:** Natália Teixeira Frota, Cid André Fidelis-de-Paula-Gomes, André Pontes-Silva, Jocassia Silva Pinheiro, Sulamizia Filomena Costa de Jesus, Gabriel Henrique Santin Apahaza, Cesário da Silva Souza, Mariana Arias Avila, Almir Vieira Dibai-Filho

**Affiliations:** 1 Department of Medicine, Universidade Federal Do Maranhão, São Luís, MA Brazil; 2 Rehabilitation Sciences, Universidade Nove de Julho, SP São Paulo, Brazil; 3 Department of Physical Therapy, Universidade Federal de São Carlos, Rod. Washington Luís, Km 235, SP 13565-905 São Carlos, Brazil; 4 Rehabilitation and Functional Performance, Universidade de São Paulo, SP Ribeirão Preto, Brazil; 5 Physical Education, Universidade Federal Do Maranhão, MA São Luís, Brazil; 6 Society, Technologies, and Public Policies, Centro Universitário Tiradentes, AL Maceió, Brazil

**Keywords:** Musculoskeletal disorders, Reproducibility of results, Functional status, Factor analysis, statistical

## Abstract

**Background:**

The Roland-Morris Disability Questionnaire (RMDQ) is one of the most used instruments to measure self-reported disability in patients with low back pain, however, the uncertainty on which version to use may lead to inadequate disability measurement and consequently, improper management of patients with chronic low back pain.

**Objective:**

To propose a short version of the RMDQ, compare it with the other short versions presented by the specialized literature, and identify the best internal structure of the RMDQ for the Brazilian population.

**Methods:**

This is a cross-sectional study in which we used confirmatory factor analysis to identify the best structure of the RMDQ. We assessed 545 participants, most of which were women, aged ≥ 30 years old, single, with mean low back pain intensity ~ 5 points, and mean pain chronicity ~ 72 months. We used lavaan and semPlot packages, with implementation of a tetrachoric matrix and the robust diagonally weighted least squares extraction method. We also used fit indices chi-square/degree of freedom, comparative fit index, Tucker-Lewis index, root mean square error of approximation, and standardized root mean squared residual. For the comparison between models, we considered the structure with the lowest values of the Akaike information criterion and Bayesian information criterion. In addition, we assessed criterion validity via Spearman’s correlation coefficient to correlate the long and short versions. In this study, the 15-item structure was created through the use of modification indices to identify redundant items (9 items were excluded).

**Results:**

RMDQ structure with one domain and 15 items and the structure with two domains and 16 items showed all fit indices with adequate values, but the one-dimensional version showed the lowest Akaike information criterion and Bayesian information criterion values. Regarding criterion validity, correlation between the RMDQ with 24 items and 15 items is adequate (rho = 0.954, *p* < 0.001).

**Conclusion:**

The RMDQ-15 is a short version of the RMDQ instrument with the most adequate internal structure and satisfactorily correlated with the long version of the instrument.

**Supplementary Information:**

The online version contains supplementary material available at 10.1186/s12891-022-05953-y.

## Introduction

The Roland-Morris Disability Questionnaire (RMDQ) is one of the most used instruments to measure self-reported disability in patients with low back pain [1]. The questionnaire was developed in the United Kingdom in 1983 and has been cross-culturally adapted for more than 30 countries, including Germany [2], Turkey [3], India [4], and Nigeria [5], but most of the adaptations failed to analyze its internal structure [6]. The Brazilian version of the RMDQ identified a valid construct through correlation with the Numerical Pain Rating Scale (NPRS) and adequate reliability [7].

The cross-cultural adaptation of the Brazilian RMDQ was conducted according to the procedures recommended by Beaton et al. [8], in which there was the translation by different translators, a consensus on the translated version, the backtranslation performed by other translators, the consensual version and the comparison between the original and the backtranslated versions [7].

Recently, a study identified a valid structure of the RMDQ with 1 domain and 24 items [9]. However, the RMDQ has a relatively high number of items to investigate disability, and item redundancy is common in long questionnaires [10]. In contrast, short questionnaires optimize clinical and scientific applicability, as they reduce the number of errors and the filling time, maintaining the same quality in the information obtained [11, 12]. Therefore, some reductions for the RMDQ have been published [13].

Study conducted by Davidson [13] evaluated three possibilities of internal structure of the RMDQ: structure with 18 items by Stratford and Binkley [14], structure also with 18 items by Williams and Myers [15], and structure with 11 items by Stroud et al. [16]. The three possibilities of internal structure showed adequate fit in the Rasch analysis. Stratford and Binkley [14] and Williams and Myers [15] used a classic test theory approach with decision rules for item reduction based on response frequency, item–item and item–total correlation, and Cronbach’s alpha; Stroud et al. [16] used an item response theory approach in developing their short version of the RMDQ.

In Brazil, there is only one short version of the RMDQ, with 16 items, validated for community-dwelling older adults with low back pain [17]. The study presented a structure with two domains: functional capacity domain, composed of items 1, 4, 6, 7, 8, 19, and 20; and mobility domain, consisting of items 3, 5, 9, 11, 14, 16, 17, 21, and 23. The items were excluded for presenting cross-loading, inadequate loading factors, and commonalities, or did not report to the latent construct. The authors used factor analysis to support this two-dimensional structure.

However, despite important scientific initiatives, no study compared the different versions of the RMDQ for the Brazilian population based on factor analysis. The uncertainty on which version to use may lead to inadequate disability measurement and consequently, improper management of patients with chronic low back pain. Thus, the present study aimed to propose a short version of the RMDQ, compare it with the short versions presented by the specialized literature, and identify the best internal structure of the RMDQ for the Brazilian population.

Our hypothesis was that there would be a more adequate RMDQ short version, and that this short version would be positively correlated with the original RMDQ. Considering the importance of assessing self-report disability in the clinical context, and that using short versions of the most used disability instruments make it easier for clinicians to apply and interpret their results in a faster way, there is a need to establish the best short-version structure of the RMDQ.

## Methods

### Study design and recruitment

This was a cross-sectional study to investigate the structural validity of the RMDQ. The methodology of this study followed the guidelines of the COnsensus-based Standards for the selection of health Measurement INstruments (COSMIN) [18]. The study was approved by the Research Ethics Committee of the institution (protocol number 3,408,949). We recruited the study sample on physiotherapy clinics in the city of Buriticupu e São Luis simultaneously (from August 2020 to July 2022). In addition, we disclosed the research via social media and messaging application (Instagram®, Facebook®, and WhatsApp®), and those who were willing to take part of the study were contacted and received a link for the Google Forms® (Mountain View, CA, USA) data collection form.

### Sample

On factor analysis, it is recommended that the sample size represents 7 times the number of items in the questionnaire [18]. As the RMDQ has 24 items, the minimum sample size was 168 participants [18]. We included native speakers of Brazilian Portuguese, of both sexes, aged ≥ 18 years, with low back pain for at least 3 months (chronic), with score ≥ 3 points on the 11-point NPRS [19]. Exclusion criteria were: tumor history, acute infections or trauma in the lumbar and pelvic region; systemic degenerative diseases; diagnosed neurological and cognitive problems; other previously diagnosed chronic pains; pregnancy.

### Evaluation tools

We used two scales in the present study: 11-points NPRS to measure pain intensity and 24-items RMDQ to measure disability. The NPRS is a scale used to quantify the pain intensity using a sequence of 11 numbers, in which 0 represents “no pain” and 10 “the worst pain imaginable” (higher the score, the greater the pain intensity), validated for Portuguese by Ferreira-Valente et al. [19]. The RMDQ was adapted and validated for the Brazilian population by Nusbaum et al. [7] and measure the disability in individuals with low back pain (reliability and construct validity tested). It consists of 24 items that describe situations experienced by people with low back pain and its score ranges from 0 to 24 points (higher the score, the greater the disability). In the face-to-face collection (cities of Buriticupu e São Luis), paper and pen were used in which the participants filled in the instruments through self-report; in the online stage, we used the Google Forms® (Mountain View, CA, USA) and extracted the database, which was controlled, making duplicate responses impossible.

### Statistical analysis

We performed descriptive analysis to present the personal and clinical variables. Quantitative variables were presented as mean and standard deviation, while qualitative variables were presented as relative and absolute frequencies.

We used confirmatory factor analysis (CFA) to identify the best structure of the RMDQ via software R Studio (Boston, MA, USA), using lavaan and semPlot packages, and with implementation of a tetrachoric matrix and the robust diagonally weighted least squares (RDWLS) extraction method [20, 21]. We considered adequate values on fit indices for the following cut-off: chi-square/degree of freedom (DF) < 3; comparative fit index (CFI) and Tucker-Lewis index (TLI) > 0.90; root mean square error of approximation (RMSEA) and standardized root mean squared residual (SRMR) < 0.08 [22, 23].

For the comparison between models, the structure with the lowest values of the Akaike information criterion (AIC) and Bayesian information criterion (BIC) was considered the most appropriate [24]. Factor loadings were considered adequate when greater than 0.40 [25].

The method of reducing the number of items of the RMDQ considered the modification indices and factor loadings (modification indices indicate redundant items in pairs). We considered redundant items to be those with a modification index value higher than 10 [26]. In each paired analysis the redundant item with the lowest factor loading was excluded, and at the end of the item exclusions, the researchers of this study approved the short version of the RMDQ.

We assessed criterion validity and considered the 24-item long version of the RMDQ as the gold standard. Thus, we used Spearman’s correlation coefficient (rho) to correlate the long and short versions, given that the data did not present a normal distribution when analyzed using the Kolmogorov–Smirnov test. Correlation magnitude > 0.70 was considered the appropriate cut-off point for criterion validity [18].

## Results

The study sample consisted of 545 participants: 428 individuals (78.5%) participated in the study by completing the online form and 117 participants (21.5%) participated face-to-face. During the face-to-face collection, an independent researcher gave the instruments to the participants to record their self-report, whose answers were obtained through individual reading and completion similar to the format of the online collection (i.e., there was no influence from the evaluator). Most participants were women, aged ≥ 30 years old, single, and physically active (Table 1). Mean low back pain intensity was ~ 5 points on NPRS and mean pain chronicity was greater than 72 months. Participants in the face-to-face data collection showed higher levels of disability in the RMDQ. However, despite the higher levels of disability, the mean RMDQ scores for both groups did not reach the RMDQ cutoff scores [27].

**Table 1:** Characteristics of patients with chronic low back pain – values presented as Mean (standard deviation) or n (%)

**Variables**	**Total sample, *n* = 545**	**Online, *n* = 428**	**Face-to-face, *n* = 117**
Age (years)	31.88 (10.35)	31.50 (10.37)	33.24 (10.20)
Sex (female)	374 (68.6%)	282 (65.9%)	92 (78.6%)
Body mass (kg)	72.19 (16.79)	73.51 (16.94)	67.34 (15.34)
Stature (m)	1.69 (0.66)	1.70 (0.74)	1.63 (0.08)
Body mass index (kg/m^2^)	25.85 (4.86)	26.06 (4.90)	25.08 (4.66)
Marital status			
Single	368 (67.5%)	251 (58,6%)	100%
Married	157 (28.8%)	157 (36.7%)	0%
Divorced	16 (2.9%)	16 (3.7%)	0%
Widower	4 (0.7%)	4 (0.9%)	0%
Scholarity			
Elementary school	38 (7.0%)	9 (2.1%)	29 (24.8%)
High school	337 (61.8%)	295 (68.9%)	42 (35.9%)
Higher education	170 (31.2%)	124 (29%)	46 (39.3%)
Physical activity (yes)	296 (54.3%)	296 (69.2%)	0%
Alcohol (yes)	187 (34.3%%)	176 (41.1%)	11 (9.4%)
Smoke (yes)	9 (1.7%)	9 (2.1%)	0%
Chronicity (months)	72.77 (74.02)	72.77 (74.02)	60.85 (51.97)
Pain (NPRS, 0–10)	5.86 (2.19)	5.86 (2.19)	5.40 (2.04)
Disability (RMDQ)			
24 items (score, 0–24)	7.07 (6.17)	5.60 (5.13)	12.45 (6.69)
15 items (score, 0–15)	3.72 (4.21)	2.64 (3.20)	7.68 (5.05)

*NPRS* Numeric Pain Rating Scale, *RMDQ* Roland Morris Disability Questionnaire

Table 2 describes the RMDQ structures that we tested, and Table 3 displays that the original 24-item RMDQ structure and the 18-item structure presented by Stratford and Binkley [14] showed two inadequate fit indices in the CFA (chi-square/DF > 3 and SRMR > 0.08). The structures suggested by Williams and Myers [15] with 18 items and by Stroud et al. [16] with 11 items showed chi-square/DF > 3.

**Table 2:** Versions of the Roland-Morris Disability Questionnaire tested in the present study

**Items**	**24 items **^**a**^	**18 items **^**b**^	**18 item **^**c**^	**11 Items **^**d**^	**15 items **^**e**^	**16 items **^**f**^
1. I stay at home most of the time because of my back	Yes	Yes	Yes	No	Yes	Yes
2. I change position frequently to try and get my back comfortable	Yes	No	No	No	No	No
3. I walk more slowly than usual because of my back	Yes	Yes	Yes	Yes	Yes	Yes
4. Because of my back, I am not doing any of the jobs that I usually do around the house	Yes	Yes	Yes	No	Yes	Yes
5. Because of my back, I use a handrail to get upstairs	Yes	Yes	Yes	Yes	Yes	Yes
6. Because of my back, I lie down to rest more often	Yes	Yes	Yes	No	No	Yes
7. Because of my back, I have to hold onto something to get out of an easy chair	Yes	Yes	Yes	Yes	No	Yes
8. Because of my back, I try to get other people to do things for me	Yes	Yes	Yes	Yes	Yes	Yes
9. I get dressed more slowly than usual because of my back	Yes	Yes	Yes	Yes	Yes	Yes
10. I only stand up for short periods of time because of my back	Yes	Yes	Yes	Yes	Yes	No
11. Because of my back, I try not to bend or kneel down	Yes	Yes	Yes	Yes	No	Yes
12. I find it difficult to get out of a chair because of my back	Yes	Yes	Yes	Yes	Yes	No
13. My back is painful almost all the time	Yes	Yes	Yes	No	Yes	No
14. I find it difficult to turn over in bed because of my back	Yes	Yes	Yes	No	No	Yes
15. My appetite is not very good because of my back pain	Yes	No	No	No	No	No
16. I have trouble putting on my socks (or stockings) because of the pain in my back	Yes	Yes	Yes	Yes	Yes	Yes
17. I only walk short distances because of my back pain	Yes	No	Yes	Yes	Yes	Yes
18. I sleep less well because of my back	Yes	Yes	Yes	No	No	No
19. Because of my back pain, I get dressed with help from someone else	Yes	No	No	No	Yes	Yes
20. I sit down for most of the day because of my back	Yes	No	No	No	Yes	Yes
21. I avoid heavy jobs around the house because of my back	Yes	Yes	Yes	Yes	No	Yes
22. Because of my back pain, I am more irritable and bad tempered with people than usual	Yes	Yes	No	No	Yes	No
23. Because of my back, I go upstairs more slowly than usual	Yes	Yes	Yes	Yes	No	Yes
24. I stay in bed most of the time because of my back	Yes	No	No	No	Yes	No

^a^Original structure [7]

^b^Structure proposed by Stratford and Binkley [13]

^c^Structure proposed by Williams and Myers [14]

^d^Structure proposed by Stroud et al. [15]

^e^Structure proposed in the present study

^f^Structure proposed by Takara et al. [16]

**Table 3:** Reduction of the Roland-Morris Disability Questionnaire items based on modification indices (MI) and factor loadings from confirmatory factor analysis

**Redundant items**	**Item description**	**MI**	**Factor loading**	**Item deleted**
**Decision 1**				
Item 2	I change position frequently to try and get my back comfortable	72.340	0.07	Item 2
Item 6	Because of my back, I lie down to rest more often		0.41	
**Decision 2**				
Item 21	I avoid heavy jobs around the house because of my back	29.984	0.48	Item 21
Item 23	Because of my back, I go upstairs more slowly than usual		0.79	
**Decision 3**				
Item 15	My appetite is not very good because of my back pain	20.767	0.96	Item 15
Item 19	Because of my back pain, I get dressed with help from someone else		0.99	
**Decision 4**				
Item 6	Because of my back, I lie down to rest more often	19.410	0.41	Item 6
Item 13	My back is painful almost all the time		0.48	
**Decision 5**				
Item 9	I get dressed more slowly because of my back	15.609	0.85	Item 14
Item 14	I find it difficult to turn over in bed because of my back		0.74	
**Decision 6**				
Item 18	I sleep less well because of my back	13.827	0.33	Item 18
Item 22	Because of my back pain, I am more irritable and bad tempered with people than usual		0.56	
**Decision 7**				
Item 11	Because of my back, I try not to bend or kneel down	12.642	0.67	Item 11
Item 15	My appetite is not very good because of my back pain		0.96	
**Decision 8**				
Item 7	Because of my back, I have to hold on to something to get out of an easy chair	10.585	0.85	Item 7
Item 12	I find it difficult to get out of a chair because of my back		0.91	
**Decision 9**				
Item 19	Because of my back pain, I get dressed with help from someone else	10.569	0.99	Item 23
Item 23	Because of my back, I go upstairs more slowly than usual		0.79	

**Fig. 1:**
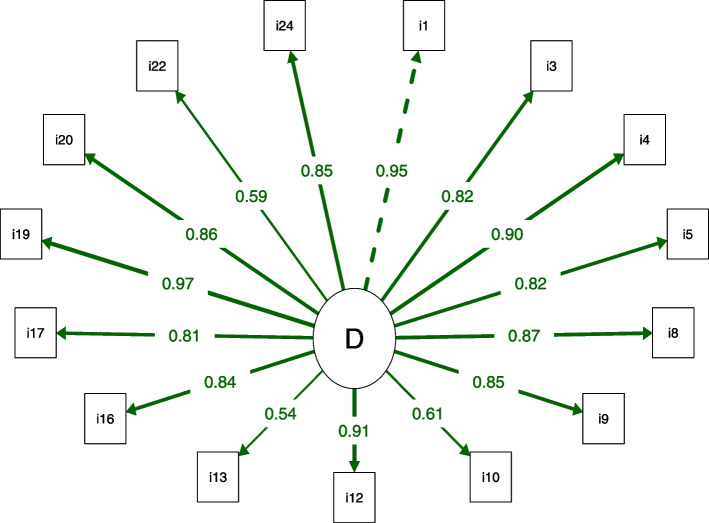
Path diagram of the 15-item Roland-Morris Disability Questionnaire. All factor loadings above 0.40. The dotted line indicates the first factor item. The thicker the line, the greater the factor loading. D: Disability

The 15-item structure proposed in the present study was created through the use of modification indices to identify redundant items. Thus, 9 items were excluded, as shown in Table 3 and Fig. 1. The RMDQ structure with one domain and 15 items and the structure with two domains and 16 items showed all fit indices with adequate values, but the one-dimensional version showed the lowest AIC and BIC values (Table 4). All of the authors of this study analyzed the RMDQ-15 and agreed with the remaining items. The Brazilian Portuguese version of the RMDQ-15 is available in Supplementary file 1.

**Table 4:** Confirmatory factor analysis of the five versions of the Roland-Morris Disability Questionnaire tested in the present study (*n* = 545)

**Models**	**Chi-square/DF**	**CFI**	**TLI**	**RMSEA (90% CI)**	**SRMR**	**AIC**	**BIC**
Model 1	3.99	0.952	0.947	0.078 (0.073, 0.083)	0.117	10,090.839	10,297.277
Model 2	3.23	0.968	0.964	0.064 (0.057, 0.071)	0.081	8629.743	8784.571
Model 3	3.02	0.972	0.969	0.061 (0.054, 0.068)	0.076	8320.427	8475.256
Model 4	3.61	0.980	0.975	0.069 (0.058, 0.081)	0.070	5056.193	5150.810
Model 5	2.38	0.988	0.986	0.050 (0.042, 0.059)	0.060	5519.272	5648.295
Model 6	2.86	0.981	0.977	0.059 (0.051, 0.066)	0.074	6332.057	6473.983

*DF* Degrees of freedom, *CFI* Comparative fit index, *TLI* Tucker-Lewis Index, *RMSEA* Root mean square error of approximation, *CI* Confidence interval, *SRMR* Standardized root mean square residual, *AIC* Akaike information criterion, *BIC* Bayesian information criterion

Model 1: Structure with one domain and 24 items [7]

Model 2: Structure with one domain and 18 items [13]

Model 3: Structure with one domain and 18 items [14]

Model 4: Structure with one domain and 11 items [15]

Model 5: Structure with one domain and 15 items

Model 6: structure with two domains and 16 items [16]

Regarding criterion validity, the correlation between the RMDQ with 24 items and 15 items is adequate (rho = 0.954, *p* < 0.001).

## Discussion

Our study identified that the Brazilian version of the RMDQ with one domain and 15 items has the best fit indices, which supports this structure as the most adequate RMDQ short version. The only study that showed a reduced version of the RMDQ in Brazil (two domains and 16 items) was carried out with participants aged 60 years or older. [17]. As in our study, the research conducted by Tanaka et al. [17] used factor analysis with correlation matrix and extraction method adequate for categorical variables. In the comparison between our structure and the structure by Tanaka et al. [17], we identified good fit indices for both, but our structure was more adequate as it presented lower AIC and BIC values.

The reduced structures of the RMDQ in English by Stratford and Binkley [14], Williams and Myers [15], and Stroud et al. [16] showed some inadequate fit indices. Several methodologies were used to reduce an instrument [14–17]. We used modification rates, factor analysis, and approval of the short version by the study authors. Considering all the proposed structures, only the following items are present in all five structures tested here: items 3, 5, 8, 9 and 16.

Regarding criterion validity, the RMDQ-15 showed a correlation magnitude above 0.95 with the long version of the instrument. This finding indicates that, even with the exclusion of 9 items, the measurement capacity of the RMDQ-15 remained very close to the original version. COSMIN indicates that correlations above 0.70 are sufficient for criterion validity. Although our study presents adequate values in the properties of measurements verified, we agree with Chiarotto et al. [1] regarding the need to evaluate other measurement properties, such as reliability, internal consistency, responsiveness, minimal clinically important difference/change, and standard error of measurement.

Disability resulting from low back pain is the main complaint of patients. This motivates them to seek professional help, and that is why the RMDQ is the most used instrument in the clinical evaluation of these patients. As such, our study will help patients, clinicians, and scientists to assess the disability of low back pain patients using less time (online or face-to-face) and with more certainty and accuracy regarding the construct that the instrument purports to measure (disability). Besides, reducing the number of items (from 24 to 15 items) also reduces the redundancy of this instrument, which will facilitate, consequently, the understanding of patients with low education, favoring the inclusion in research on low back pain and facilitating specific treatments for them (because the specificity of a treatment depends on the accuracy of the assessment).

The study has limitations that must be considered. Most of the sample in our study had mild disability related to low back pain, despite the inclusion criterion of a minimum NPRS score of three points. Therefore, we recommend further studies to test RMDQ-15 in patients undergoing clinical treatment with higher degrees of disability, as well as other RMDQ-15 measurement properties, e.g., construct validity, reliability, internal consistency, responsiveness, minimal clinically important difference/change, and standard error.

## Conclusion

The RMDQ-15 is a short version of the RMDQ instrument with the most adequate internal structure and satisfactorily correlated with the long version of the instrument.

## Supplementary Information

**Additional file 1. **

## Data Availability

The data and materials in this paper are available from the corresponding author on request.

## Abbreviations

AIC: Akaike Information Criterion
BIC: Bayesian Information Criterion
CFA: Confirmatory Factor Analysis
CFI: Comparative fit index
COSMIN: COnsensus-based Standards for the selection of health Measurement INstruments
DF: Degree of freedom
NPRS: 11-Item Numeric Pain Rating Scale
RDWLS: Robust Diagonally Weighted Least Squares
RHO: Spearman’s Correlation Coefficient
RMSEA: Root Mean Square Error of Approximation
RMDQ: Roland-Morris Disability Questionnaire
RMDQ-15: 15-Item Roland-Morris Disability Questionnaire
SRMR: Standardized Root Mean Squared Residual
TLI: Tucker-Lewis index
