# Supplementary material for: 15-item Roland-Morris Disability Questionnaire (RMDQ-15): structural and criterion validity on patients with chronic low back pain
Source: BMC Musculoskelet Disord. 2022 Nov 12;23:978. doi: 10.1186/s12891-022-05953-y (PMC9652881; doi:10.1186/s12891-022-05953-y)
Supplement: Supplementary file 1 — Additional file 1. [file 12891_2022_5953_MOESM1_ESM.pdf]

English version of the 15-item Roland-Morris Disability Questionnaire (RMDQ-15)

When your back hurts, you may find it difficult to do some of the things you normally do. This list contains some sentences that people have used to describe themselves when they have back pain. When you read them, you may find that some stand out because they describe you today. As you read the following list, think of yourself today. When you read a sentence that describes you today, put a tick against it. If the sentence does not describe you then leave the space blank and go on to the next one. Remember, only tick the sentence if you are sure that it describes you today.

		Sentences
1	( )	I stay at home most of the time because of my back.
3	( )	I walk more slowly than usual because of my back.
4	( )	Because of my back, I am not doing any jobs that I usually do around the house.
5	( )	Because of my back, I use a handrail to get upstairs.
8	( )	Because of my back, I try to get other people to do things for me.
9	( )	I get dressed more slowly than usual because of my back.
10	( )	I only stand up for short periods of time because of my back.
12	( )	I find it difficult to get out of a chair because of my back.
13	( )	My back is painful almost all of the time.
16	( )	I have trouble putting on my sock (or stockings) because of the pain in my back.
17	( )	I can only walk short distances because of my back pain.
19	( )	Because of my back pain, I get dressed with the help of someone else.
20	( )	I sit down for most of the day because of my back.
22	( )	Because of back pain, I am more irritable and bad tempered with people than usual.
24	( )	I stay in bed most of the time because of my back.

Versão brasileira do Roland-Morris Disability Questionnaire com 15 itens (RMDQ-15)

Quando suas costas doem, você pode encontrar dificuldade em fazer algumas coisas que normalmente faz. Esta lista possui algumas frases que as pessoas têm utilizado para se descreverem quando sentem dores nas costas. Quando você ler estas frases, pode notar que algumas se destacam por descrever você hoje. Ao ler a lista de frases, pense em você hoje. Quando ler uma frase que descreve você hoje, assinale-a. Se a frase não descreve você, então deixe o espaço em branco e siga para a próxima frase.

		**Frases**
1	( )	Fico em casa a maior parte do tempo por causa de minhas costas.
3	( )	Ando mais devagar que o habitual por causa de minhas costas.
4	( )	Por causa de minhas costas, eu não estou fazendo nenhum dos meus trabalhos que geralmente faço em casa.
5	( )	Por causa de minhas costas, eu uso o corrimão para subir escadas.
8	( )	Por causa de minhas costas, tento conseguir com que outras pessoas façam as coisas por mim.
9	( )	Eu me visto mais lentamente que o habitual por causa de minhas costas.
10	( )	Eu somente fico em pé por períodos curtos de tempo por causa de minhas costas.
12	( )	Encontro dificuldades em me levantar de uma cadeira por causa de minhas costas.
13	( )	As minhas costas doem quase que o tempo todo.
16	( )	Tenho problemas para colocar minhas meias (ou meia calça) por causa das dores em minhas costas.
17	( )	Caminho apenas curtas distâncias por causa de minhas dores nas costas.
19	( )	Por causa de minhas dores nas costas, eu me visto com ajuda de outras pessoas.
20	( )	Fico sentado(a) a maior parte do dia por causa de minhas costas.
22	( )	Por causa das dores em minhas costas, fico mais irritado(a) e mal humorado(a) com as pessoas do que o habitual.
24	( )	Fico na cama a maior parte do tempo por causa de minhas costas.
